# SERIES: eHealth in primary care. Part 6: Global perspectives: Learning from eHealth for low-resource primary care settings and across high-, middle- and low-income countries

**DOI:** 10.1080/13814788.2023.2241987

**Published:** 2023-08-24

**Authors:** Margot Rakers, Steven van de Vijver, Paz Bossio, Nic Moens, Michiel Rauws, Millicent Orera, Hongxia Shen, Cynthia Hallensleben, Evelyn Brakema, Nick Guldemond, Niels H. Chavannes, María Villalobos-Quesada

**Affiliations:** a Department of Public Health and Primary Care, Leiden University Medical Centre, Leiden, the Netherlands; b National eHealth Living Lab (NELL), Leiden, the Netherlands; c Amsterdam Health & Technology Institute, Amsterdam, the Netherlands; d Universidad Nacional de Jujuy, San Salvador de Jujuy, Argentina; e Africa eHealth Foundation, Veenendaal, the Netherlands; f Cass, San Francisco, CA, USA; g PharmAccess Foundation, Amsterdam, the Netherlands; h School of Nursing Guangzhou, Guangzhou Medical University, Guangdong, China; i Sechenov University Moskva, Moskva, Russia

**Keywords:** eHealth, low resource settings, primary care, digital health, Low- and Middle-Income Countries, High-Income Countries

## Abstract

**Background:**

eHealth offers opportunities to improve health and healthcare systems and overcome primary care challenges in low-resource settings (LRS). LRS has been typically associated with low- and middle-income countries (LMIC), but they can be found in high-income countries (HIC) when human, physical or financial resources are constrained. Adopting a concept of LRS that applies to LMIC and HIC can facilitate knowledge interchange between eHealth initiatives while improving healthcare provision for socioeconomically disadvantaged groups across the globe.

**Objectives:**

To outline the contributions and challenges of eHealth in low-resource primary care settings.

**Strategy:**

We adopt a socio-ecological understanding of LRS, making LRS relevant to LMIC and HIC. To assess the potential of eHealth in primary care settings, we discuss four case studies according to the WHO ‘building blocks for strengthening healthcare systems’.

**Results and discussion:**

The case studies illustrate eHealth’s potential to improve the provision of healthcare by i) improving the delivery of healthcare (using AI-generated chats); ii) supporting the workforce (using telemedicine platforms); iii) strengthening the healthcare information system (through patient-centred healthcare information systems), and iv) improving system-related elements of healthcare (through a mobile health financing platform). Nevertheless, we found that development and implementation are hindered by user-related, technical, financial, regulatory and evaluation challenges. We formulated six recommendations to help anticipate or overcome these challenges: 1) evaluate eHealth’s appropriateness, 2) know the end users, 3) establish evaluation methods, 4) prioritise the human component, 5) profit from collaborations, ensure sustainable financing and local ownership, 6) and contextualise and evaluate the implementation strategies.

KEY MESSAGES

- Low Resource Settings (LRS) are found in Low- Middle- and High-Income Countries.
- Case studies can inform eHealth initiatives in LRS, facilitating knowledge interchange.
- eHealth can contribute in LRS to primary care’s daily practice and system-related elements but user-related, technical, evaluation, financial and regulatory challenges must be anticipated or addressed.

## Introduction

eHealth promises to improve health and healthcare systems. It has also been proposed that eHealth has the potential to support low-resource primary care systems by, for example, improving service provision, facilitating the doctor-patient relationship and the shared decision-making process, and promoting self-management. However, relevant barriers to successful development, implementation, and adoption are often reported [1,2].

This article is part of a series about eHealth in primary care [2–6], and focuses on eHealth in low-resource primary care settings. Low-resource settings (LRS), as defined in this article (Box 1), can be found in high-income countries (HIC) and low- and middle-income countries (LMIC). We take that approach because lessons can be learnt from developing and applying eHealth in low-resource primary care settings independently of the countries’ income level. This can contribute to optimising eHealth-enabled services in general and improve the health of socioeconomically disadvantaged groups across the globe. Although other publications have addressed eHealth in LRS, the relevance of LRS in LMIC and HIC is rarely addressed.

The previous articles of this series comprehensively describe the challenges that primary care faces [2–6]. Primary care systems at a global scale need to adapt to these challenges to ensure the quality of care. This need is even more pressing in LRS where anticipating and effectively addressing these issues is key for ensuring the quality of care and optimising available resources [7,8]. Acute or chronic healthcare emergencies, such as the COVID-19 pandemic and the climate and refugee crises, have shown us that addressing these challenges is imperative worldwide [6,9].

As presented in the fourth article of this series, the implementation of eHealth in clinical practice needs to adapt to the healthcare system, patients, all stakeholders and society in general [5]. It also must be adequately incorporated into pre-established and carefully considered processes, training, and routines in the medical practice. These requisites are equally important in low-resource primary care settings and must be achieved with limited resources. Learning from previous initiatives and promoting knowledge interchange can increase the chances of success. When working across borders or translating interventions developed elsewhere to one’s own context, the heterogeneity of primary care systems worldwide must be considered. For example, the differential role of community or public health components, organisational structures, regulations and standards should be evaluated.

In this article, we analyse the potential of eHealth in primary care settings based on four case studies and according to the ‘building blocks for strengthening healthcare systems’ (WHO) [9]. The analysis includes the contributions and challenges of the use cases and examples of similar initiatives in HIC and LMIC. Finally, we categorise the identified challenges and summarise the lessons learnt in six recommendations.

## Case study analysis of eHealth in low-resource primary care settings

**Figure 1.**
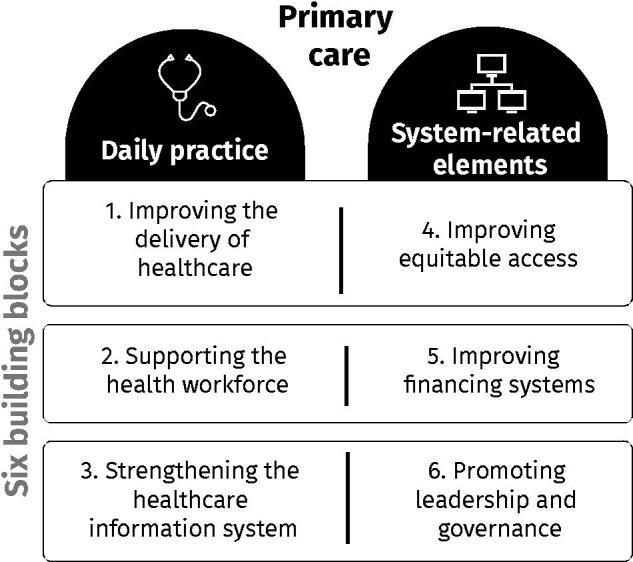
Building blocks of healthcare systems. According to the ‘WHO Framework for Action’, six ‘building blocks’ are necessary to achieve the goals of healthcare systems: improving health and health equity, responding to the expectations of the population, ensuring social and financial risk protection, and achieving this through the efficient use of available resources [9].

The ‘WHO Framework for Action’ is used as a guide to identify and discuss the contributions and challenges of eHealth in low-resource primary care settings [9]. This framework proposes six ‘building blocks’ necessary for the good functioning of healthcare systems Figure 1). The first three building blocks related to ‘everyday primary care practice’ are addressed separately by analysing three independent case studies, while the last three building blocks, which correspond to ‘system-related elements’ of healthcare, are illustrated by one case study. The analysis of the case studies provides evidence of the successful implementation of eHealth in low-resource primary care settings in LMIC and HIC and can be used as guidance for similar applications or contexts.

### Improving daily primary care practice in LRS through eHealth

#### Building block 1. Improve the delivery of healthcare – case study 1: Cass

**Figure 2.**
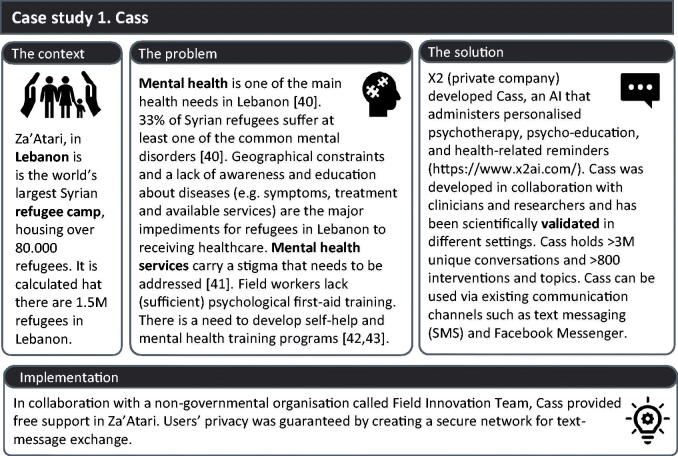
Case study 1, Cass, an AI chatbot that offers mental health services to refugees in Lebanon.

Automated and semi-automated systems, such as for telemonitoring and electronic health record management have been gaining ground in delivering primary care [2,10,11]. Cass (Figure 2) exemplifies how eHealth can facilitate mental health services, specifically in the world’s largest Syrian refugee camp in Lebanon. In this context, AI-generated chats that provide self-help advice have overcome the lack of sufficient resources, limited staff and language barriers.

#### Cass’ challenges and solutions

General AI-related challenges such as transparency, accountability, and bias were addressed from the project’s onset; for example, the company developed a generic ethical guideline for its mental health AI systems [12]. Problems with connectivity, including internet coverage, were addressed by providing offline and toll-free SMS text messages through local providers. Cass depends on accessibility to enabling technologies, i.e. mobile phones but it has been made flexible to avoid compatibility issues. Visibility of the services was improved by training local nurses to introduce Cass during their visiting rounds. Furthermore, the heterogeneous target patient population makes it necessary to constantly monitor and improve the system to avoid misunderstandings, bias or inaccuracies related to, for example, different cultural backgrounds and languages. The strategy to address these issues is user-centred and includes the participation of local translators in the system’s development and iterative content improvement based on the end-user’s experience [10,11].

#### Other contributions of eHealth to the delivery of healthcare

(Semi-)automated eHealth solutions, such as Cass, can be applied in contexts where (structural) healthcare services are lacking or insufficient, for example, in assisting refugees and asylum seekers [13]. (Semi-)automated eHealth systems in primary care have also been applied to test and follow up on sexually transmitted infections, disproportionately affecting underserved populations and ethnic minorities in LMIC and HIC [14]. They have also shown positive results in a cross-country Latin American study aimed at the primary prevention of the progression of hypertension with eHealth [15,16]. Similarly, (semi-)automated systems have been implemented for triage in the face of the COVID pandemic [17,18]. In the Netherlands, for example, a mobile and web-based application for after-hours self-triage is offered in five languages, improving accessibility in areas with multicultural neighbourhoods (>100.000 households) [19].

#### Building block 2. Support the health workforce – case study 2: Molulo Telemedicine Programme (TP)

**Figure 3.**
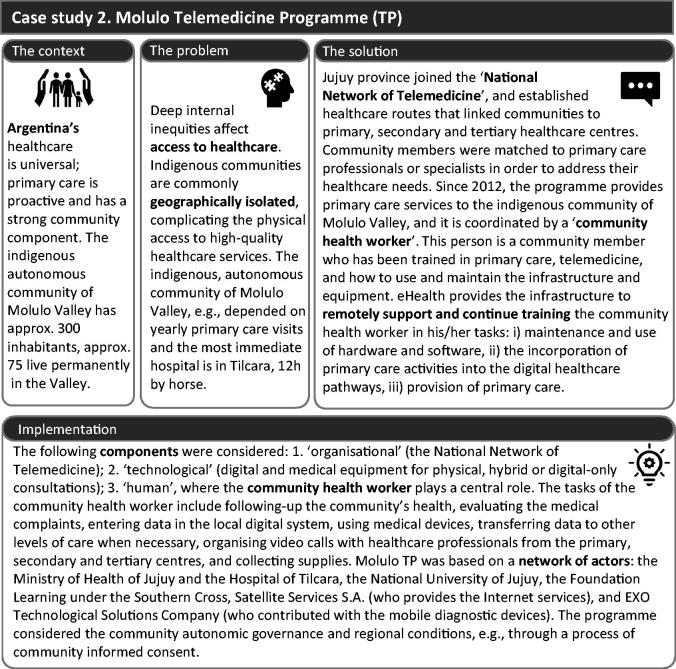
Case study 2, Molulo Telemedicine Programme, programme that also supports the health workforce in remote indigenous communities in Argentina.

Telemedicine is probably the most well-known eHealth application and is a tool for making primary care accessible to remote communities. The Molulo TP in Argentina (Figure 3) has shown that eHealth can positively impact healthcare delivery and support the health workforce, but implementation and sustainability are complex. In the case of the indigenous community of Molulo Valley, a geographically isolated community, the telemedicine infrastructure has served as viable means to train and support the healthcare staff on the ground.

#### Molulo TP’s challenges and solutions

Since 2019 the programme has been under constant evaluation and improvement. One strategy has been coordinating the care services of several isolated communities in the region to optimise resources. The healthcare worker’s role, which greatly depends on his/her digital literacy, has been key to success. Financial insecurity is a current challenge resulting from the programme’s reliance on several public and private parties whose commitment may vary over time and a lack of readily available resources in emergencies (e.g. system failure or equipment damage because of environmental conditions, COVID-19). Additionally, implementation research and formal evaluation are challenging to fund and carry out.

#### Other contributions of eHealth to supporting the health workforce in primary care

Evidence shows that telemedicine has successfully provided primary care to other isolated communities like the First Nation communities in Canada and circumpolar regions [20,21]. eHealth applications in LRS have also been used to support the workforce in specific medical areas such as family planning and screening for sexually transmitted infections in Tanzania, mental health in Afghanistan and ophthalmology in China [14,22,23]. Artificial Intelligence systems are also being researched as a tool to improve the scheduling of community health worker visits [24].

#### Building block 3. Strengthening the healthcare information system – case study 3: AfyaPro

**Figure 4.**
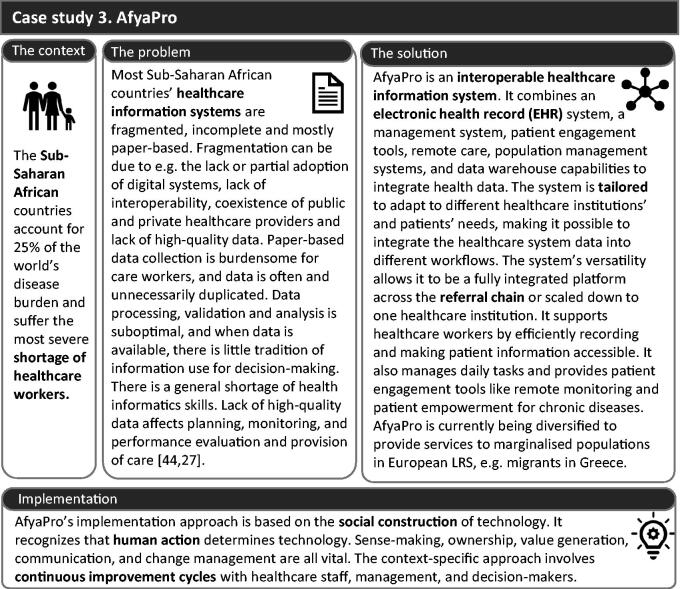
Case study 3, AfyaPro, interoperable electronic patient systems used in Malawi, Zimbabwe, Ghana and Kenya.

Healthcare systems are often fragmented, failing to integrate the different levels of care efficiently. Patient-centred healthcare information systems, such as AfyaPro (Figure 4), can contribute to overcoming these issues in LRS [25,26]. AfyaPro is an example for countries with developed health systems that shows the advantages of collaborating in developing and adopting integrated solutions.

#### AfyaPro’s challenges and solutions

Although AfyaPro has been deployed in several countries, it must continuously improve to overcome upcoming challenges such as insufficient eHealth and ICT skills, limited training opportunities and limited local technical support. Creating new roles and modifying practices have been necessary to ensure the system is used successfully. Furthermore, contextual influences in a complex sociotechnical system challenge the system’s evaluation. Currently, retrospective evaluation studies are being carried out [25]. Finally, regular power cuts have sometimes led to patient data loss, a factor that is difficult to control.

#### Other contributions of eHealth to healthcare information systems in primary care

Other health information systems have been reported to improve reporting and health data integration and assist clinical decision-making in LRS [27,28]. Additionally, these systems can protect patients’ privacy and promote patients’ self-management and autonomy. This is particularly relevant in the face of rising non-communicable diseases worldwide [29,30]. Besides improving patients’ health, quality of care, and healthcare workers’ experience, these healthcare information systems can play an important role in public health surveillance and research activities [31].

### Improving the ‘system-related elements’ of healthcare

#### Building blocks 4–6. Improving equitable access and financing systems and promoting leadership and governance – case study 4: M-TIBA

**Figure 5.**
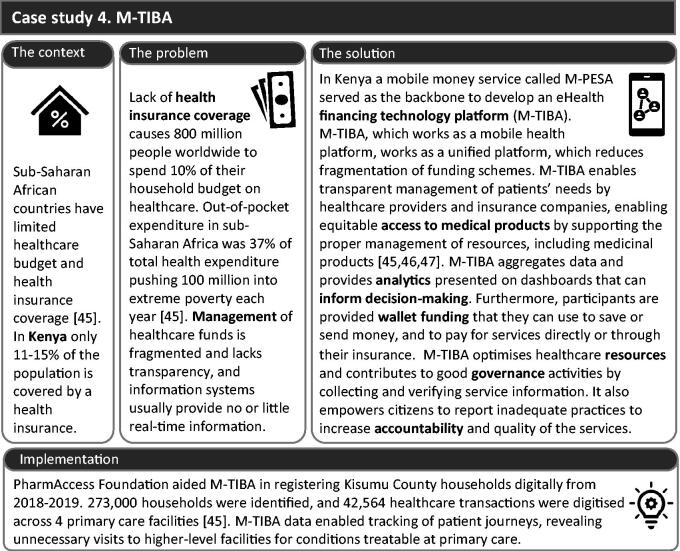
Case study 4, M-TIBA, a mobile health platform to provide a transparent financing system in Kenya.

eHealth can also contribute to the healthcare’s ‘system-related elements’ [9]. The support given to general practitioners at this level may be less evident but no less important than the previous three building blocks. Without them, the primary care practice is incapable of functioning. M-TIBA, a mobile health financing platform (Figure 5), illustrates how eHealth can improve equitable access, financing systems, leadership, and governance.

#### M-TIBA’s challenges and solutions

M-TIBA’s developers emphasised that interoperability between healthcare systems and insurance companies is often a barrier. This problem was addressed *via* an application programming interface (API). The lack of clear national policy standards is also a challenge, requiring the development of an overarching health policy to ensure the inclusion of M-TIBA as a digital innovation in different settings. Additionally, the upfront costs of M-TIBA may impede healthcare systems from implementing this digital financing system. Other implementation barriers, such as poor connectivity, inappropriate infrastructure, and limited support, motivated the development of cloud-based systems. Lastly, low (eHealth) literacy has also been a barrier to embracing the technology on the ground. To improve this situation, M-TIBA’s team and other stakeholders have trained healthcare providers to use the platform and implementation is carried out closely with healthcare professionals.

#### Other contributions of eHealth to improving equitable access and financing systems, and promoting leadership and governance in primary care

Other eHealth applications have been reported to contribute to healthcare’s ‘system-related elements’, such as supporting the systems that ensure equitable access to medical products. For example, digital systems using drones as the delivery mechanism have improved supply chains and can quickly respond to different needs. This approach has assisted areas affected by natural disasters, unexpected accidents in difficult access areas, urban contexts, and the COVID-19 pandemic. They can also combine features for surveying damaged areas or identifying mosquito breeding sites remotely [32,33]. Regarding leadership and governance, eHealth has supported governments in improving data exchange and quality of care by using digital technology management systems. For example, in South Africa and Uganda, eHealth has facilitated the planning, distribution and managing of vaccination programmes [34].

## Categorisation of challenges in LRS and recommendations

### Challenges

To optimise eHealth interventions, it is necessary to identify the present challenges and barriers, or those more likely to occur. This can help the teams on the ground to prepare and anticipate or to minimise and properly address these challenges. Based on the analysis of the case studies and accompanying examples, five types of challenges were identified (Box 2).

### Recommendations

In light of these challenges, six recommendations (summarised in Box 3) specific to low-resource primary care settings were drawn. These recommendations complement previously reported findings in LRS, such as reported best practices for scaling eHealth in LMIC [35].

#### Determine whether eHealth is the appropriate solution to unmet healthcare needs or to improve the provision of care

Providing care *via* eHealth should always be evaluated upfront to ensure it is the right means to achieve the desired goals. eHealth may be more efficient than traditional care, for example, benefiting groups who are not accessing or unable to access traditional care, such as in the case of sexually transmitted diseases and underserved and/or isolated populations (see Molulo TP and Cass). It is also possible that eHealth as a stand-alone service may be the best short-term option but blended care may be the most appropriate strategy in the long run (see Cass). However, it is important to consider that eHealth may not be the best solution.

#### Get to know your end users

eHealth should consider the individual needs (usability and acceptability), socio-economic context (including family and other social structures), (eHealth) literacy, and the needs of all involved parties. Identify feasible eHealth solutions based on local population needs, through inviting professionals to be involved in developing and implementing eHealth programmes for future adoption and appropriate use. Because success ultimately depends on the end-user’s readiness to adopt the solution, an open and informed dialogue among stakeholders at all stages is key (see Molulo TP and M-TIBA). We argue that eHealth can strongly contribute to improving equity by including those who have been left behind. This can be achieved *via* inclusive design and by facilitating the acquisition of skills followed by evaluating the end-user’s capability to use eHealth (see Molulo TP).

#### Consider the appropriate evaluation methods

After confirming that eHealth is the appropriate solution (first recommendation) and engaging end-users (second recommendation), we recommend implementing evidence-based solutions (see Cass and M-TIBA). If the eHealth solution is being developed or no evidence exists, evaluation of effectiveness and implementation research should be carried out (see Molulo TP and AfyaPro). Randomised controlled trials may not always be possible, so alternative methods should be considered, such as pre-post-test design or observational studies.

#### Prioritise the human component

Not only a human-centred approach is necessary for the development and implementation of eHealth, but the support and generation of user skills should also be prioritised. For example, eHealth literacy can be improved by establishing an implementation plan that includes training and support for end-users. Remote communications can be used to deliver care and can also be used as a platform to train the healthcare workforce (see Molulo TP). Another possibility is strengthening eHealth training in education programmes for students and professionals to improve general (eHealth) literacy (see M-TIBA).

#### Profit from collaborations between public and private partners and ensure sustainable financing and local ownership

Sustainable financing for eHealth is challenging in LRS. Engaging public and private parties can help develop, deploy and fund eHealth interventions, but it may bring financial uncertainty (see Cass, Molulo TP and M-TIBA). Planning from the programme’s onset on achieving sustainable financing should be a priority, which can be facilitated by fostering local ownership.

#### Contextualise and evaluate implementation strategies when introducing eHealth to a new setting

The effectiveness and suitability of eHealth can vary significantly depending on the context (see Cass). Thus, the implementation pathway should be flexible and adaptable, and stepwise frameworks should be utilised during the development and implementation stages (see AfyaPro).

## Conclusion

LRS have been systematically associated with LMIC, preventing, at least in part, rich knowledge exchange with initiatives in HIC. Based on our own experience, there is little internalisation of the learnings of eHealth projects in LMIC when developing solutions for underserved or marginalised populations in HIC. The case studies presented here have demonstrated that user-centricity, stakeholder engagement, appropriate planning and evaluation, sustainable collaborations and financing are key elements that should be considered for developing and implementing eHealth interventions globally. However, user-related, technical, financial, regulatory and evaluation challenges remain. Identifying these challenges can help prepare eHealth interventions to avoid or overcome these issues. The lessons learnt from eHealth in LRS might also be helpful around the globe when designing and implementing eHealth to address acute or chronic healthcare emergencies, such as the harmful effects of environmental pollution and climate change on health, conflicts and large-scale population movements, and the quick spread of infectious viruses and global pandemics. By outlining the contributions and challenges of eHealth in low-resource primary care settings, we aim to strengthen the link between LMIC and HIC. The recommendations are helpful to operationalise the lessons learned from the case studies and their analysis, and we hope that they will be used to guide the future development and implementation of eHealth in LRS or improve ongoing eHealth initiatives.
